# Assessing the Effectiveness and Safety of Combination Diuretic Therapy in Heart Failure: A Systematic Review and Meta-Analysis

**DOI:** 10.7759/cureus.72118

**Published:** 2024-10-22

**Authors:** Rebecca Ayoti, Zahid Khan, Gideon Mlawa, Animesh Gupta

**Affiliations:** 1 Cardiology, University of South Wales, Treforest, GBR; 2 Acute Medicine, Mid and South Essex NHS Foundation Trust, Southend on Sea, GBR; 3 Cardiology, Bart’s Heart Centre UK, London, GBR; 4 Cardiology and General Medicine, Barking, Havering and Redbridge University Hospitals NHS Trust, London, GBR; 5 Cardiology, Royal Free Hospital, London, GBR; 6 Internal Medicine and Diabetes and Endocrinology, Barking, Havering and Redbridge University Hospitals NHS Trust, London, GBR; 7 Acute Internal Medicine, Southend University Hospitals NHS Trust, Southend on Sea, GBR; 8 Acute Internal Medicine/Intensive Care, Barking, Havering and Redbridge University Hospitals NHS Trust, London, GBR

**Keywords:** combination diuretics in heart failure, decompensated heart failure, heart failure advancements, heart failure and medical education, heart failure hospitalization, heart failure management programmes, heart failure prognosis, heart failure with reduced ejection fraction, mortality in acute heart failure, valvular heart disease

## Abstract

Heart failure remains a significant clinical challenge due to the difficulty in managing refractory fluid overload. Combination diuretic strategies, involving various combinations, such as metolazone and furosemide, have been proposed to improve diuresis and patient outcomes. This systematic review and meta-analysis aimed to evaluate the efficacy and safety of combination therapies. A comprehensive search of PubMed, Cochrane Library, Embase, and Web of Science identified 61 articles, of which seven randomised controlled trials met the inclusion criteria. Data on mortality, hospital readmission rates, symptom improvement, electrolyte imbalances, renal function, and adverse events were extracted and analysed. The risk of bias was assessed using the established guidelines. The analysis revealed that combination diuretic therapies significantly reduced body weight (P=0.001) but did not significantly impact mortality (RR: 0.99, 95% CI: 0.90-1.09) or hospital readmission rates (RR: 1.05, 95% CI: 0.98-1.12) compared to placebo. Adverse effects, particularly electrolyte imbalances, such as hypo and hypernatraemia and hypokalaemia, and renal function deterioration were noted in the combined diuretic group. In contrast, serious adverse events were observed more in the placebo group. The mean difference of the Kansas City Cardiomyopathy Questionnaire (KCCQ) score was 2.43 (95% CI: 0.95-3.92). The risk ratio for hospital readmission was 1.05 (95% CI: 0.98-1.12), which was statistically non-significant. We used fixed-effect models for most variables due to less heterogeneity between the studies, and the corresponding I^2^ values were <50% for most variables. Funnel plots indicated minimal publication bias, although some heterogeneity was observed. Comparisons with other studies in the literature, such as the DAPA-HF and EVEREST trials, supported these findings but also highlighted the need for individualised treatment approaches. Combination diuretic therapies effectively manage fluid overload and reduce body weight in patients with heart failure but do not significantly affect mortality or hospital readmission rates. The potential for adverse events, particularly electrolyte imbalances and renal function deterioration, underscores the need for careful monitoring and personalised treatment plans. Future research should focus on optimising diuretic combinations and dosing strategies to enhance their safety and efficacy. These findings align with the current guidelines emphasising individualised treatment in heart failure management and highlight the importance of integrating combination diuretics into a comprehensive care plan to improve patient outcomes.

## Introduction and background

Pulmonary congestion is a common cause of presentation among individuals requiring hospital admission for acute heart failure (AHF) [1]. Therefore, one of the primary goals of in-hospital care for these patients is to alleviate congestion signs and symptoms (also known as decongestion) [2-4]. Unfortunately, after hospital admission, adequate decongestion is frequently not obtained, and this has been associated with increased mortality and hospitalisation rates for heart failure (HF) [5-6]. The persistence of congestion post-admission necessitates the exploration of targeted strategies to enhance decongestion and improve patient outcomes. Currently, loop diuretics are the cornerstone of treatment for patients with HF who present with congestion [7-8]. However, the effectiveness of loop diuretics can diminish over time due to progressive disease and the development of diuretic resistance, necessitating higher doses, which can lead to adverse effects, such as electrolyte imbalances, renal dysfunction, and worsening cardiovascular outcomes [9]. Consequently, there is growing interest in the use of combination diuretic therapies, which involve the concurrent use of different classes of diuretics to optimise fluid management while minimising the adverse effects associated with high-dose monotherapy [8,10]. Combination diuretic therapies employ various classes of diuretics, including loop diuretics, thiazide diuretics, and mineralocorticoid receptor antagonists (MRAs). By targeting multiple sites of renal sodium reabsorption, these combinations aimed to enhance diuresis and achieve better decongestion outcomes. The rationale behind this approach is to overcome the limitations of single-agent therapy, which often fails to provide adequate decongestion in advanced HF cases.

Rationale for evaluating combination diuretic therapies

Combination diuretic strategies involving the concurrent use of different classes of diuretics, such as loop diuretics, thiazide diuretics, and MRAs, to target multiple sites of renal sodium reabsorption and enhance diuresis while minimizing adverse effects associated with high-dose monotherapy are being evaluated as potential methods of achieving adequate decongestion among HF patients. There are several challenges to evaluating the effectiveness of combination diuretic therapies for HF. First, the lack of standardised protocols or guidelines for these therapies results in significant variability in dosing regimens and drug combinations across studies. This variability makes it difficult to compare results and draw definitive conclusions. Additionally, individual patient characteristics such as renal function, electrolyte status, and comorbidities can significantly influence the response to diuretic therapy, complicating the interpretation of the study results. For instance, patients with compromised renal function may experience different outcomes than those with normal renal function, making it challenging to establish a one-size-fits-all approach. Moreover, there are concerns regarding the potential synergistic effects of combination diuretic therapy on electrolyte imbalance, renal function decline, and neurohormonal activation. These potential adverse effects underscore the importance of rigorous evaluations to assess the effectiveness and safety of these therapies. The complexity of managing HF with combination diuretics necessitates a thorough understanding of how these therapies interact with various physiological parameters [11-12]. Given the current scarcity of high-quality evidence and the clinical uncertainty surrounding combination diuretic therapies for HF, there is a pressing need for a comprehensive systematic review. The goal of this systematic review was to inform evidence-based clinical decision-making and help clinicians optimise fluid management strategies in patients with HF. By identifying the most effective and safest diuretic combinations, healthcare providers can tailor treatments according to individual patient needs, ultimately improving the overall management of HF. Additionally, this review aimed to highlight areas where further research is needed to guide future studies to address unresolved questions and improve clinical practice.

## Review

Methodology

This was a systematic review and therefore adhered to the Preferred Reporting Items for Systematic Reviews and Meta-Analyses (PRISMA) guidelines [12]. A comprehensive literature search was conducted using the electronic databases PubMed and Cochrane Database of Systematic Reviews from May 1, 2023, to May 31, 2023. In addition, the systematic review register PROSPERO was searched. The search strategy involved a combination of Medical Subject Headings (MeSH) terms and keywords related to AHF, diuretic therapies, and study design. The search terms included “acute heart failure" OR "acute decompensated heart failure" AND "diuretics" OR "combination therapy" AND "randomized controlled trial" OR "quasi-experimental. “To ensure thoroughness, a manual search of the reference lists of studies meeting the PICO criteria and inclusion/exclusion criteria was performed. Additional database searches were conducted to include existing guidelines and recommendations on diuretic therapies in HF. The study selection process included articles evaluating the effects of combination diuretic therapies in patients hospitalized with AHF.

Two reviewers independently assessed the titles and abstracts of articles for relevance, duplication, and inclusion. The full texts of potentially relevant studies were retrieved and assessed for inclusion based on the predefined Population, Intervention, Comparison, and Outcome (PICO) model and inclusion/exclusion criteria. Any disagreements were resolved by consensus or by a third-party adjudicator, and eligibility criteria were established before the selection process. Articles were eligible if they were systematic reviews, meta-analyses, or randomised controlled studies and included a predefined PICO model. Articles on animals or non-human subjects and studies involving hospitalized patients for reasons other than decongestion were excluded. Specifically, the criteria were as follows. The target population in this study were patients hospitalized with AHF requiring intravenous diuretics. Patients hospitalized for other management that did not involve decongestion were also excluded. Studies of combination diuretic therapy were included. Studies without combination diuretic therapy were excluded, and studies comparing combination diuretic therapies to placebo or monotherapy with diuretics were included. Studies not reporting the effectiveness and safety outcomes, including mortality, hospital readmission rates, symptom improvement, electrolyte abnormalities, and renal function, were excluded. Studies with outcomes unrelated to effectiveness and safety were excluded. We included studies that were randomised controlled trials (RCTs) and quasi-experimental studies, whereas case studies, letters, case reports, editorials, and comments were excluded from the analysis.

Publication type

Only peer-reviewed articles published in English were included, as shown in Table 1.

**Table 1:** Inclusion and exclusion criteria based on the PICO framework PICO, Population, Intervention, Comparison, and Outcome

Criteria	Inclusion	Exclusion
Population	Patients hospitalized with acute heart failure for decongestion	Patients with acute heart failure hospitalized for other management that does not include decongestion
Intervention	Combination diuretic therapies	No combination diuretic therapy
Comparison	Placebo or monotherapy with diuretics	No comparison group
Outcome	Effectiveness and safety outcomes, including mortality, hospital readmission rates, symptom improvement, electrolyte abnormalities, and renal function	Outcomes not related to effectiveness and safety
Study design	Randomised controlled trials and quasi-experimental studies	Articles, case studies, letters, case reports, editorials, comments
Publication type	Peer-reviewed articles published in English	Studies not published in the English language were excluded due to potential costs associated with professional translational services

Full-text articles of potentially relevant studies were retrieved and assessed for inclusion based on eligibility criteria. Data extraction was performed using the systematic review software COVIDENCE.

Risk-of-bias assessment

**Figure 1:**
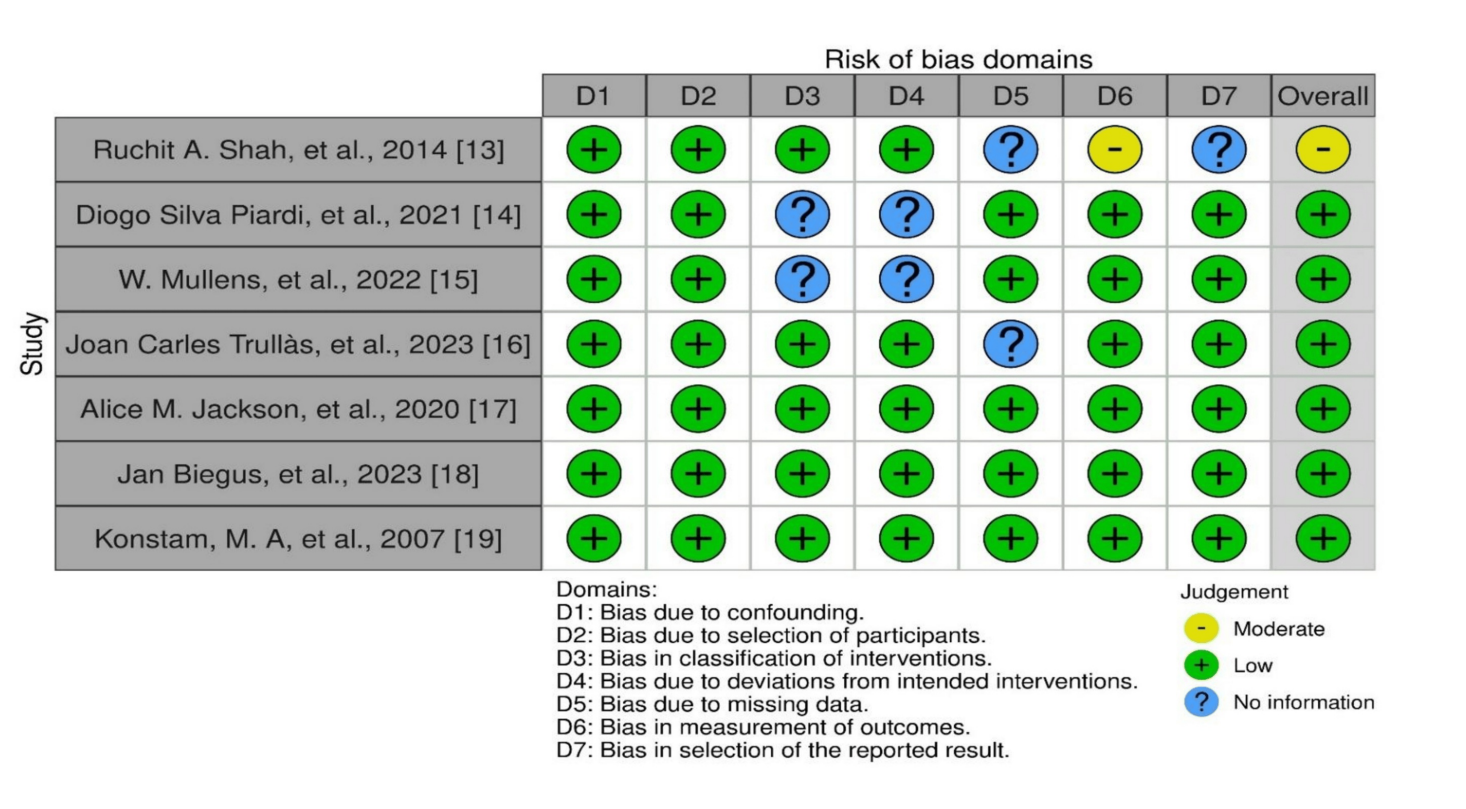
Traffic light chart for the risk-of-bias assessment

**Figure 2:**
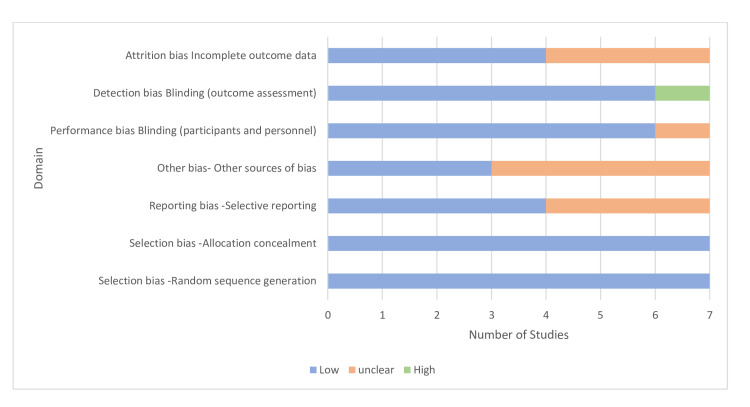
Risk-of-bias assessment for included studies

Two reviewers independently assessed the risk of bias using the appropriate tools for different types of studies. For RCTs, the Cochrane Risk of Bias tool was used to critically appraise methodological quality. For quasi-experimental studies, the Risk of Bias in Non-randomized Studies of Interventions (ROBINS-I) tool was used. The results of these assessments were compared to determine inter-rater reliability. Each tool consists of various domains used to evaluate the risk of bias and the overall quality of the studies. The studies were rated accordingly based on the presence of flaws and accuracy. This rigorous process helps identify high-quality studies and ensures the reliability of the review. The quality assessments of all studies that met the PICO and the inclusion criteria are reported in Figure 1. The summary of the risk of bias assessment for all seven selected studies is illustrated in Figure 2.

Results

Study Selection

**Figure 3:**
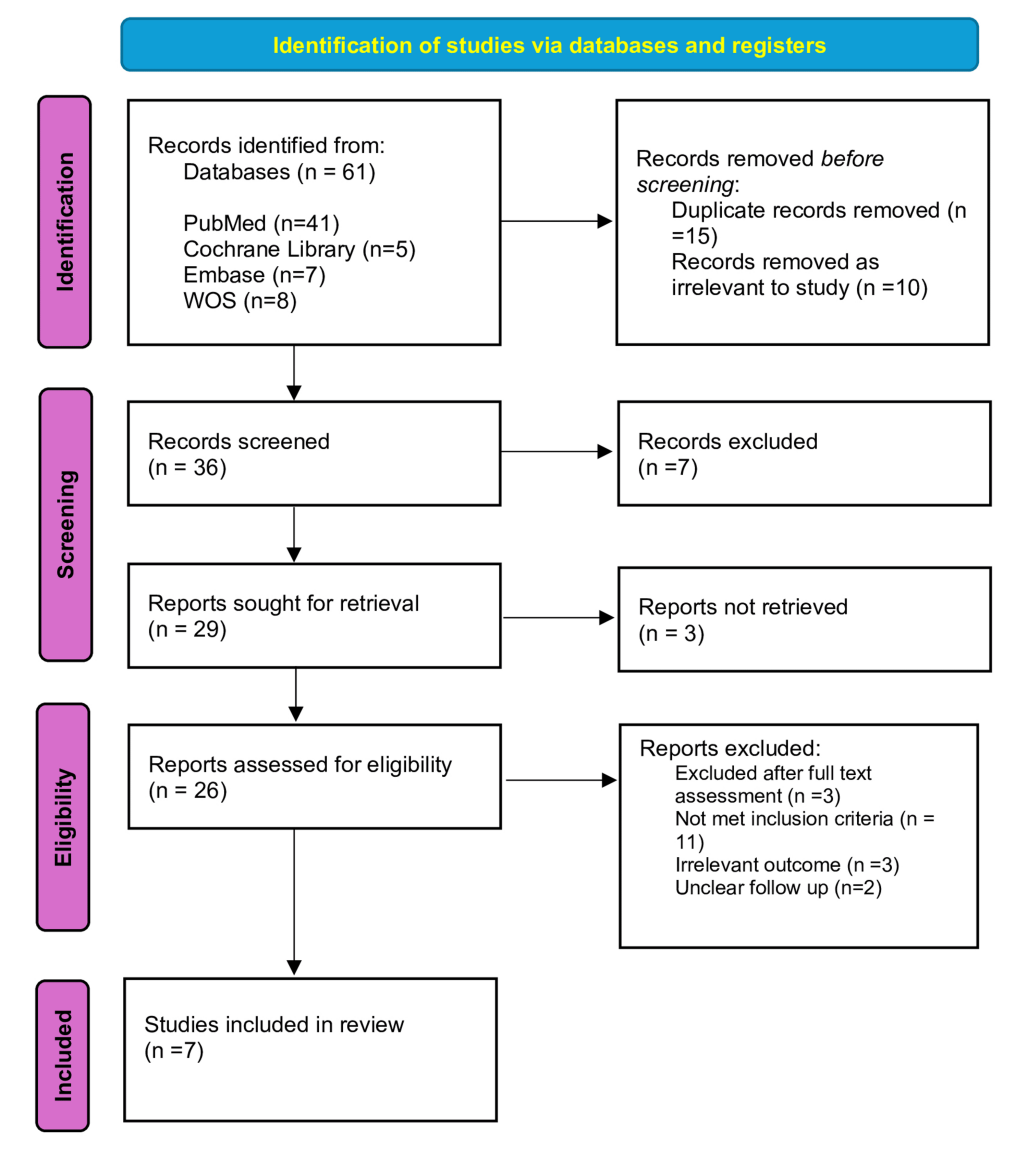
PRISMA flow diagram PRISMA, Preferred Reporting Items for Systematic Reviews and Meta-Analyses

Four databases (PubMed, Cochrane Library, Embase, and Web of Science) provided 61 articles. In total, 15 duplicate records were removed, and 10 records were removed because they were irrelevant to the study. Following the full-text screening of the remaining articles, seven RCTs made up the final selection were included in the systematic review and meta-analysis. Figure 3 shows the Preferred Reporting Items for Systematic Reviews and Meta-Analyses (PRISMA) flow diagram representing the search strategy [12].

Characteristics of incorporated RCTs

Table 2 provides a summary of the study characteristics of the RCTs that were included in this review. The sample size in the obtained articles ranges from 51 to 4,133 participants, and the average age of the participants was between 58 and 83 years [13-19]. Most studies reported that most of the population among the participants were male. Here, all the articles mainly focused on acute decompensated HF, but one study investigated HF with reduced ejection fraction [17]. Most of the studies focused on loop diuretics, particularly furosemide (FSM). Two studies investigated thiazide diuretics (hydrochlorothiazide [HCTZ]) [14,16]. One study compared empagliflozin (a sodium-glucose cotransporter-2 [SGLT2] inhibitor) to a placebo, alongside standard diuretic therapy [18]. Another study compared tolvaptan (a vasopressin receptor antagonist) to a placebo [19].

**Table 2:** A summary of study characteristics of the RCTs HCTZ, hydrochlorothiazide; RCT, randomised controlled trial

Author, Year	Study Design	Sample Size	Age	Gender	Type of Heart Failure	Types of Diuretics	Dosages	Duration of Treatment	Primary Outcome	Secondary Outcome
Shah et al., 2014 [13]	Prospective, randomised controlled trial	N=90. Group 1: 30; group 2: 30; group 3: 30	Mean age: 58.22 ± 15.45 years	Male: 66 (73.3%)	Acute decompensated heart failure	Loop diuretics (furosemide), intravenous	Group 1: Furosemide 100 mg/24h + Dopamine 2.5 mg/kg/min; Group 2: Furosemide 100 mg/24h; Group 3: Furosemide 100 mg/24h	48 hrs	Negative fluid balance at 24 hrs	Negative fluid balance at 48, 72, 96 hrs; trend of serum sodium, potassium, blood urea, creatinine; hospital stay duration; clinical outcomes at 30 days
Piardi et al., 2021 [14]	Randomised, single-centre, double-blind, placebo-controlled trial	N=51. HCTZ group: 26; placebo group: 25	Mean age: 64 years	HCTZ group: 69% male; placebo group: 48%	Acute decompensated heart failure	Furosemide + HCTZ; placebo	HCTZ: 50 mg or placebo (oral)	3 days	Daily weight change over 3 days	Change in creatinine, need for vasoactive drugs, change in natriuretic peptides, congestion score, dyspnoea scale, thirst scale, length of stay, in-hospital mortality, hypernatremia, hypokalaemia, renal function, need for haemodialysis, ventricular arrhythmias
Mullens et al., 2022 [15]	Multicentre, parallel-group, double-blind, placebo-controlled trial	N=519. Acetazolamide group: 259; placebo group: 260	Placebo: 78.5±8.8; acetazolamide: 77.9 ±9.0	Placebo: 59.6% male; acetazolamide: 65.6% male	Acute decompensated heart failure	Acetazolamide with loop diuretic	Acetazolamide: 500 mg once daily	3 days	Successful decongestion	Death from any cause or rehospitalisation for heart failure; safety and adverse events
Trullàs et al., 2023 [16]	Multicentre, prospective, randomised, double-blind, placebo-controlled trial	N=230. Placebo: 116; HCTZ: 114	Mean age: 83 years	48% women	Acute decompensated heart failure	HCTZ with intravenous furosemide	HCTZ: 25-100 mg daily based on glomerular filtration rate; Furosemide: 80 mg/day	5 days	Changes in body weight and patient-reported dyspnoea at 72 hrs	Diuretic response metrics, mortality/rehospitalisations at 30 and 90 days, safety outcomes (renal function/electrolytes)
Jackson et al., 2020 [17]	Randomised, double-blind, placebo-controlled trial	N=736 (no diuretic). Total with diuretic: 3,880	Average age: 66.1 years across groups	Female: 23.6% overall	Heart failure with reduced ejection fraction	Loop diuretics (azosemide, bumetanide, furosemide, torsemide); combination of loop and thiazide-like diuretics	Mean: 57.0 ± 94.4 mg Furosemide-Equivalent; Median: 40 mg (range 20-80 mg)	Nil	Dapagliflozin reduced the risk of the primary endpoint across diuretic subgroups	Improvement in symptoms and treatment toleration, consistency across diuretic subgroups, unchanged diuretic dose during follow-up, no difference in mean diuretic dose post-randomisation
Biegus et al., 2023 [18]	Multinational, multicentre, randomised, double-blind trial	N=530. Empagliflozin: 265; placebo: 265	Empagliflozin: 71 (62–78); placebo: 70 (59–78)	Empagliflozin: 67.5% male; placebo: 64.9%	Acute heart failure	Furosemide (intravenous and oral), torasemide, bumetanide	Empagliflozin: 10 mg once daily; furosemide: 40 mg intravenous; torasemide: 20 mg; bumetanide: 1 mg	90 days	Weight loss, WL adjusted for mean daily loop diuretic dose	Area under the curve of change in N-terminal pro-B-type natriuretic peptide levels, haemoconcentration, clinical congestion score
Konstam et al., 2007 [19]	Randomised, double-blind, placebo-controlled study	N=4,133. Tolvaptan: 2,072; placebo: 2,061	Tolvaptan: 65.9 years (SD = 11.7); placebo: 65.6 years (SD = 12.0)	Tolvaptan: 73.4% male; placebo: 75.4%	Acute decompensated heart failure	Furosemide (loop diuretic)	Low-dose: equal to outpatient dose; high-dose: 2.5 times outpatient dose; continuous infusion; intermittent bolus	60 days	Death from cardiovascular causes or first hospitalisation for heart failure	Composite of cardiovascular death or hospitalisation, incidence of cardiovascular mortality, clinical worsening of heart failure

Study findings on clinical outcomes and adverse events

The seven RCTs included in this analysis provided a comprehensive overview of the clinical outcomes and adverse events associated with various diuretics in HF management. These results indicate that while some diuretics improve symptoms and patient-reported outcomes, they may also pose risks related to electrolyte imbalances and renal function deterioration. Table 3 summarises the clinical outcomes and adverse events.

**Table 3:** Comparison of clinical outcomes and adverse events in diuretic therapy trials. HCTZ, hydrochlorothiazide; KCCQ, Kansas City Cardiomyopathy Questionnaire

Author, Year	Mortality	Hospital Readmission Rates	Symptom Improvement	Electrolyte Abnormalities	Renal Function	Other Adverse Events
Shah et al., 2014 [13]	0 in dopamine infusion group; 2 in bolus group; 1 in infusion group	9 in infusion + dopamine group; 8 in bolus group; 7 in infusion group	None reported	No statistically significant difference in serum sodium and potassium levels (p > 0.05)	No statistically significant difference in blood urea and serum creatinine levels (p > 0.05)	None reported
Piardi et al., 2021 [14]	In-hospital mortality: HCTZ 3.8%, Placebo 0% (p = 1.00)	None reported	Slight improvement in dyspnoea and congestion scores with HCTZ, but not statistically significant	Hypernatremia: HCTZ 0%, placebo 4.8%. Hypokalaemia: HCTZ 3.8%, placebo 4.5%. Increase in creatinine: HCTZ 58%, placebo 41% (p = 0.05)	Significant increase in creatinine levels in the HCTZ group compared to placebo	Ventricular arrhythmias: HCTZ 3.8%, placebo 4%. Haemodialysis: HCTZ 3.8%. placebo 0% (p = 1.00)
Mullens et al., 2022 [15]	Death: acetazolamide 29.7%, placebo 27.8% (HR 1.07; 95% CI, 0.78-1.48)	Acetazolamide: 18.4%; placebo: 17.4%	None reported	Hypokalaemia: acetazolamide 5.5%, placebo 3.9%	Renal safety endpoint: acetazolamide 2.7%, placebo 0.8%. Doubling of serum creatinine: acetazolamide 0.8%, placebo 0%. ≥50% decrease in GFR: acetazolamide 1.6%, placebo 0.4%	Serious adverse events: acetazolamide 48.0%, placebo 47.9%. Cardiovascular adverse events: acetazolamide 44.1%, placebo 47.1%
Trullàs et al., 2023 [16]	30-day mortality: HCTZ 9.6%, placebo 6.0%. 90-day mortality: HCTZ 20.2%, placebo 16.4%	HCTZ: 37.7%; placebo: 34.5%	Slight improvement in dyspnoea in the HCTZ group at 72 and 96 hours	Sodium ≤ 130 mmol/L: HCTZ 8.8%, placebo 5.2%. Sodium ≤ 125 mmol/L: HCTZ 2.6% placebo 1.7%. Potassium ≤ 3.5 mmol/L: HCTZ 44.7%, placebo 19.0%. Potassium ≤ 3.0 mmol/L: HCTZ 40.6%, placebo 16.1%	Impaired renal function: HCTZ 46.5%, placebo 17.2%. Increase in serum creatinine > 26.5 μmol/L: HCTZ 46.5%, placebo 17.2% (p < 0.001)	None reported
Jackson et al., 2020 [17]	Placebo: 289 deaths. Dapagliflozin: 245 deaths (across all diuretic doses)	None reported	Significant improvement in KCCQ total symptom score with dapagliflozin across all diuretic doses	None reported	Slight increase in creatinine levels with dapagliflozin, but fewer renal adverse events	None reported
Biegus et al., 2023 [18]	None reported	None reported	Significant weight loss and slight improvement in congestion score with empagliflozin	None reported	None reported	None reported
Konstam et al., 2007 [19]	Composite outcomes of death and unscheduled urgent clinic visit: orthodema score 0: 50% event rate; orthodema score 3-4: 68% event rate	Rehospitalisation: Orthodema score 1-2: 52% event rate	Weight loss from baseline to discharge: 12.2 lbs (low-grade orthodema); 14.2 lbs (high-grade orthodema at baseline); 14.6 lbs (high-grade orthodema at discharge)	None reported	None reported	None reported

Statistical analysis

We used RevMan statistical software version 5.4.0 (The Cochrane Collaboration, Copenhagen, Denmark) to perform meta-analysis on data extracted from these studies. Fixed or random effect models was used according to the Mantel-Haenszel model to assess study heterogeneity for most variables, and funnel plots were used for detecting publication bias. We calculated the risk ratio or mean difference for variables with corresponding confidence intervals, as shown in the forest plots. The risk ratio with 95% confidence interval was used as a measure of the treatment effect for the various variables such as electrolyte imbalance, mortality, adverse events, and serious adverse events. The mean difference was measured for continuous variables, and a p-value of less than or equal to 0.05 was considered statistical significance for each outcome of interest. I^2^ statistics was used to determine statistical heterogeneity for each outcome. I^2^ values of 50% or less showed mild or moderate heterogeneity, whereas I^2^ values of 75% showed high level of heterogeneity. A fixed effect model was used for outcome of interest with I^2^ values less than 50%, and random effect model was used for outcome of interest with I^2^ value greater than 50%.

Main outcomes

Mortality

**Figure 4:**
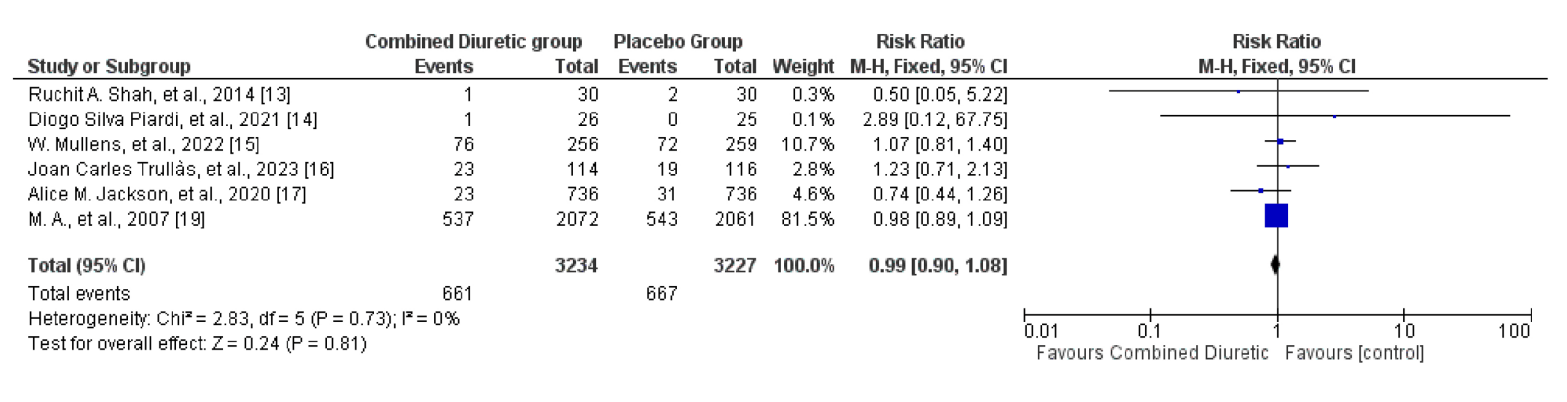
Forest plot showing mortality with 95% confidence interval and Mantel–Haenszel ratio for the combined diuretic and placebo groups.

Combination diuretic regimens did not show a statistically significant difference in mortality risk compared to the placebo group, as indicated by the overall risk ratio of 0.99 and the 95% confidence interval of 0.90 to 1.09 (Figure 4). The included studies were consistent with each other (I² = 0%), and the overall effect was not statistically significant (P = 0.85). A fixed effect model was used due to the lack of heterogeneity in the included studies. This suggests that, based on the available evidence, combined diuretic therapy does not significantly affect the mortality risk outcome compared with placebo. Concerning the individual agents based on the studies included in the review, FSM and HCTZ did not significantly impact mortality rates when compared with placebo. In contrast, acetazolamide was associated with increased mortality rates. Dapagliflozin, on the other hand, showed a reduction in deaths across all dosage groups compared with placebo.

**Figure 5:**
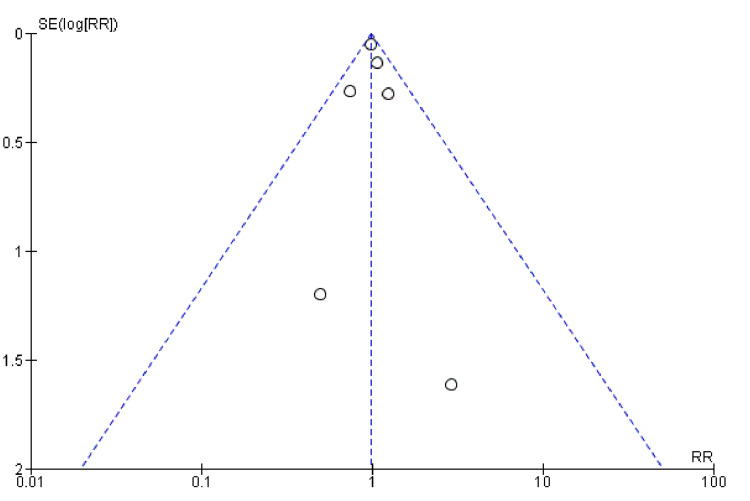
Funnel plots showing mortality for the combined diuretic and placebo groups

There did not seem to be a significant publication bias, as the funnel plot (Figure 5) was relatively symmetrical. Some level of heterogeneity is indicated by the spread of studies, which is typical of meta-analyses. Overall, the funnel plot suggests that the meta-analysis results on mortality are robust, with a minimal indication of publication bias, although some heterogeneity among the studies was present.

Hospital Readmission Rates

**Figure 6:**
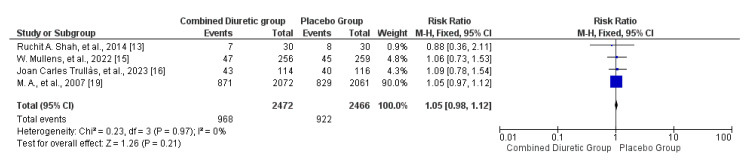
Forest plots of hospital readmission showing the comparison between the combined diuretic and placebo groups

**Figure 7:**
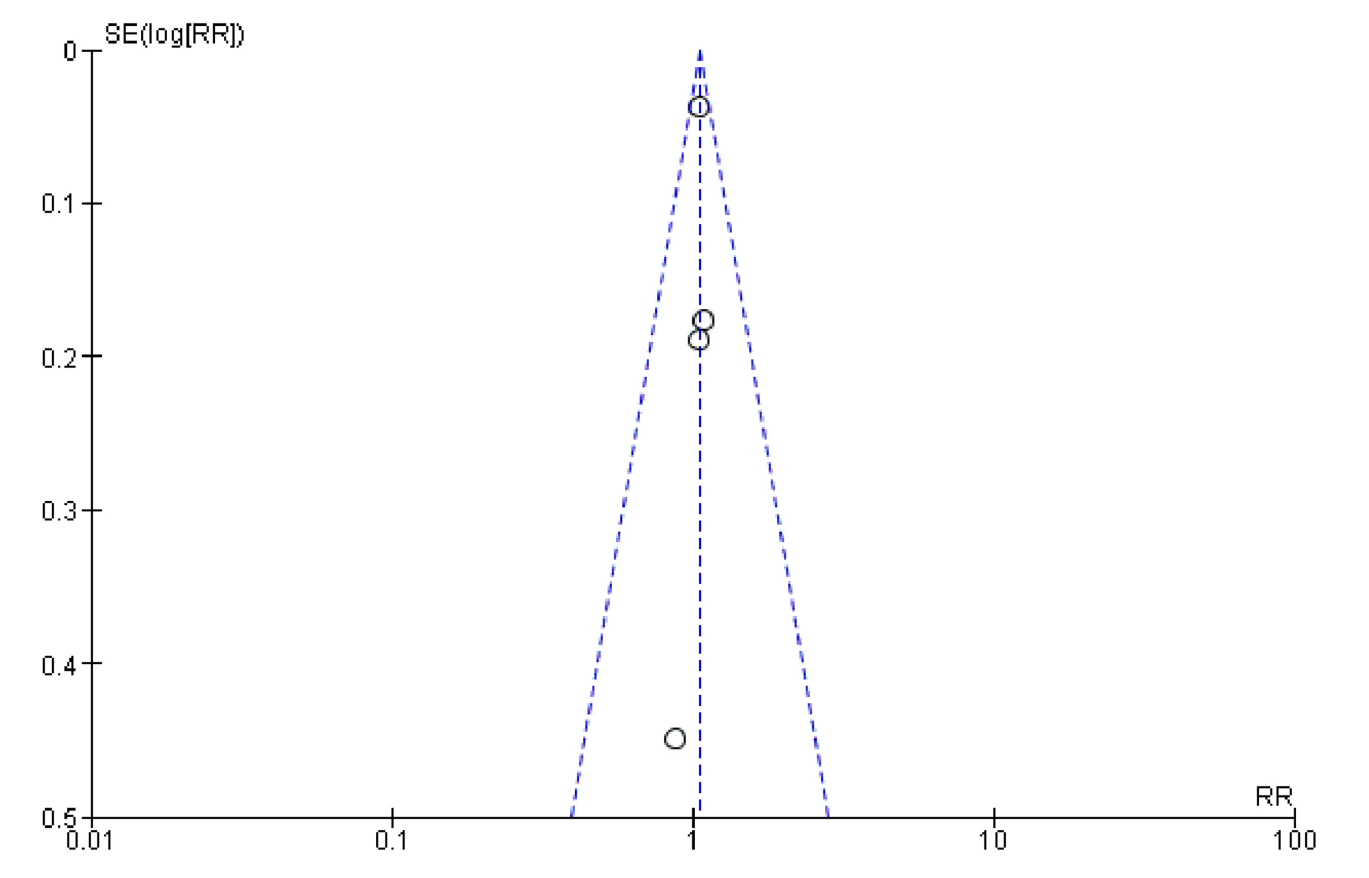
Funnel plots of hospital readmission showing confidence interval and Mantel–Haenszel ratio for the combined diuretic and placebo groups

Most studies reported no significant differences between diuretic treatment and placebo in terms of hospital readmission rates. The forest plot of hospital readmission rates (Figure 6) demonstrates that the combined diuretic group does not have a statistically significant difference in hospital readmission rates compared to the placebo group, as indicated by the overall risk ratio of 1.05 (95% CI, 0.98-1.12). Additionally, the funnel plot (Figure 7) did not show significant asymmetry, suggesting minimal or no publication bias.

Symptom Improvement

**Figure 8:**
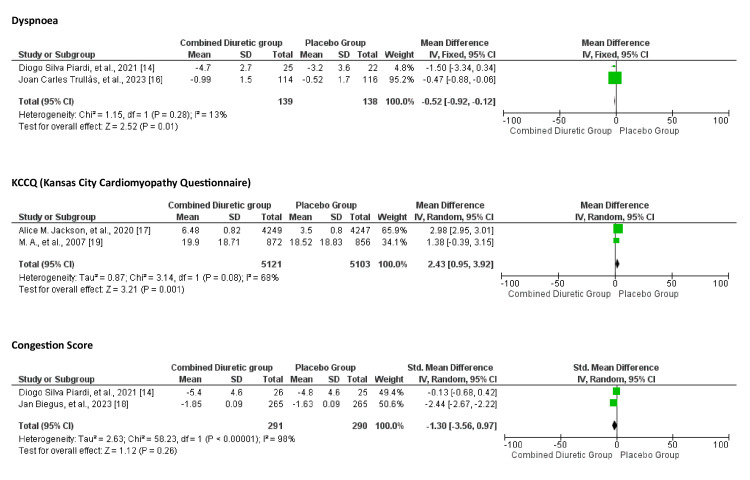
Forest plot showing comparison between the combined diuretics and placebo groups for dyspnoea, KCCQ, and congestion scores KCCQ, Kansas City Cardiomyopathy Questionnaire

**Figure 9:**
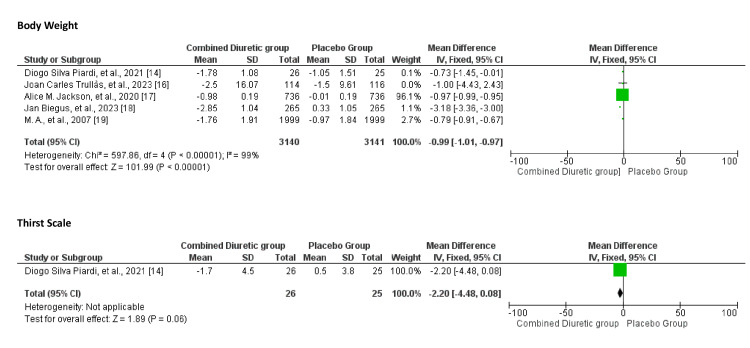
Forest plot showing body weight and thirst level comparison between the diuretics and placebo groups

The effect of combination diuretics on symptom improvement compared to placebo is shown in Figures 8, 9. Combination diuretics significantly reduced body weight (p=0.001). Additionally, there were trends towards improvement in dyspnoea (p=0.09) and thirst scale scores (p=0.06), but these did not reach statistical significance. These results suggest that while combination diuretics are effective in reducing body weight, their impact on other symptoms such as dyspnoea, KCCQ score, congestion, and thirst was not statistically significant, although some trends towards improvement were observed.

**Figure 10:**
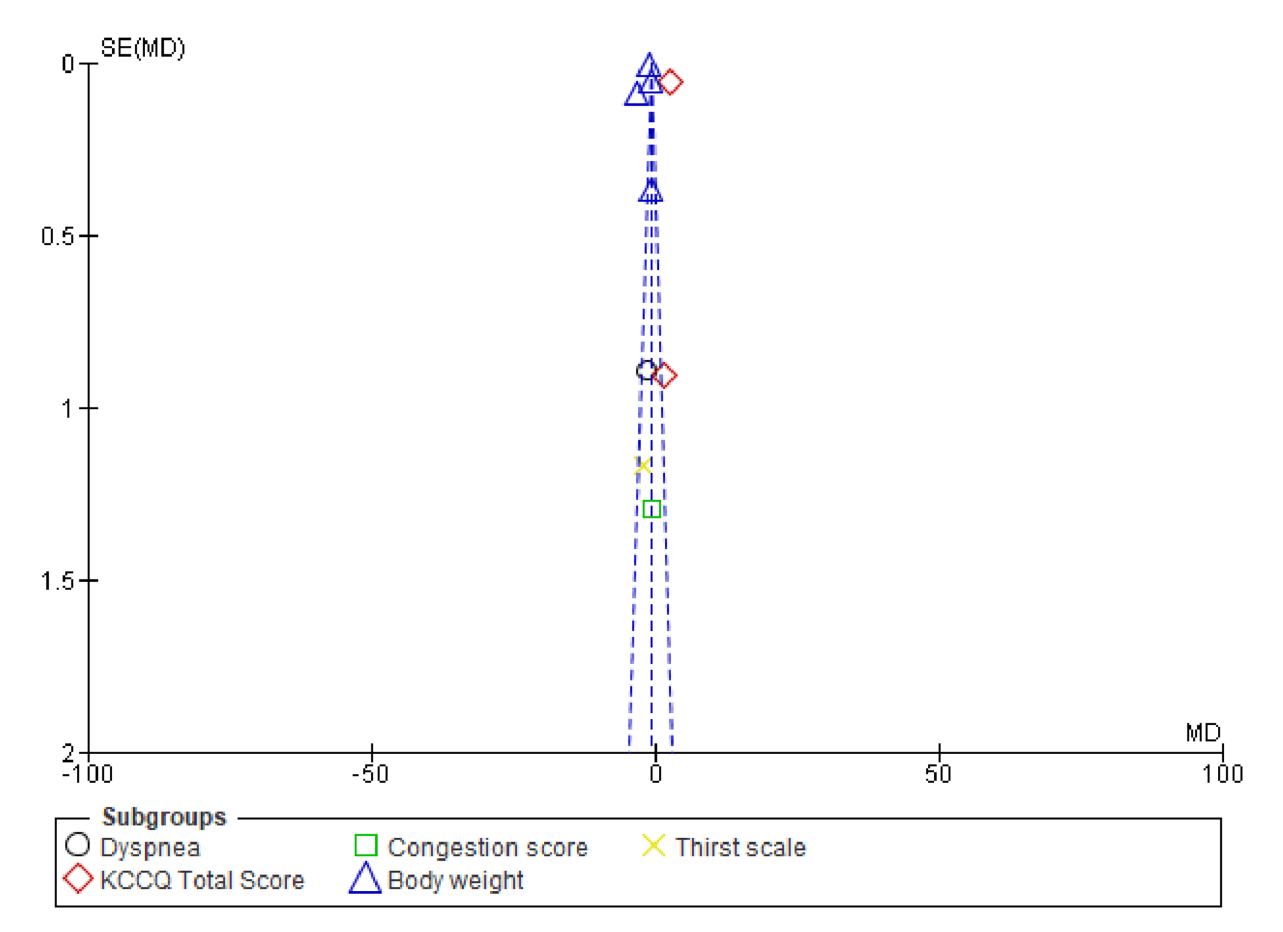
Funnel plots of symptom improvement showing confidence interval and Mantel–Haenszel ratio for the combined diuretic and placebo groups

The funnel plot (Figure 10) does not show significant asymmetry, suggesting minimal to no publication bias. The studies appear to be relatively homogeneous, with a consistent pattern around the central line.

Electrolyte Abnormalities

**Figure 11:**
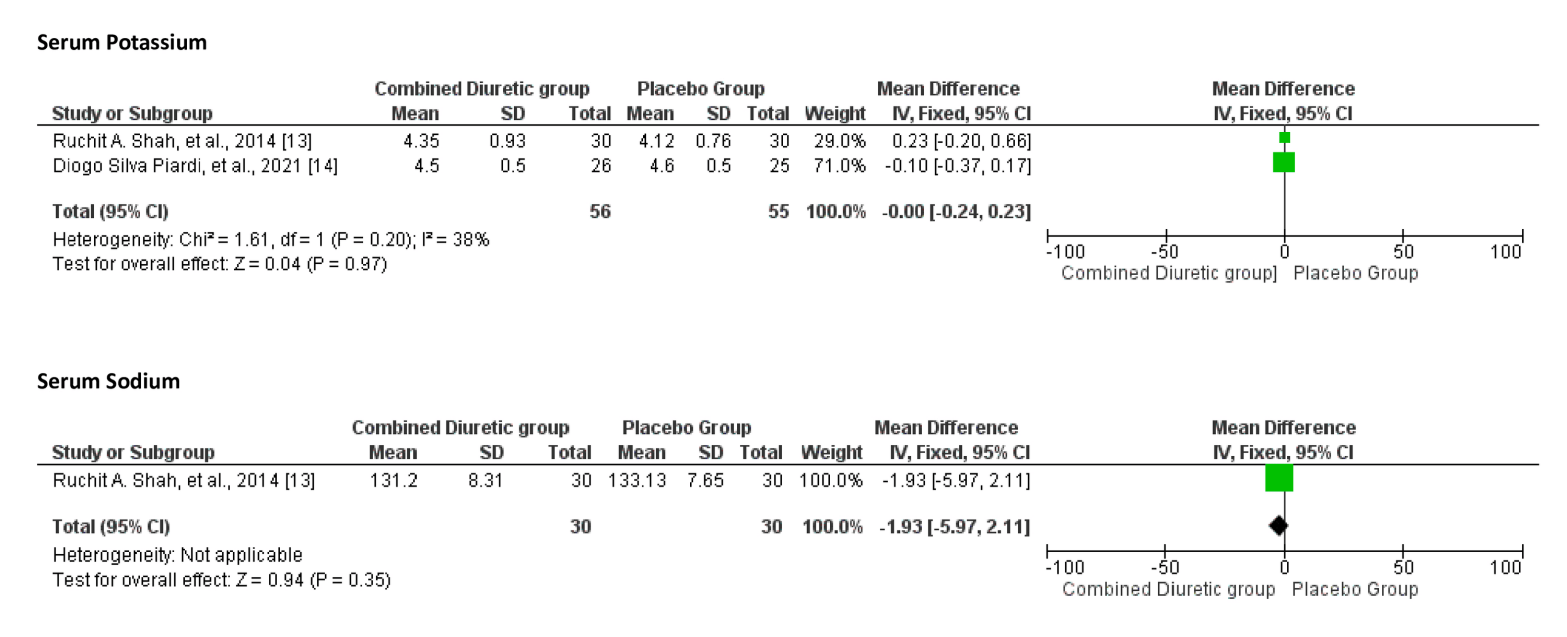
Forest plot showing electrolyte abnormalities for the combined diuretic and placebo groups

Figure 11 shows the overall electrolyte imbalances compared with placebo in combination with diuretics. The meta-analysis suggests that the use of combined diuretics does not significantly impact serum potassium or sodium levels compared with placebo. The evidence for serum sodium was limited to one study, and the heterogeneity in the results for serum potassium indicates some variability among the included studies.

**Figure 12:**
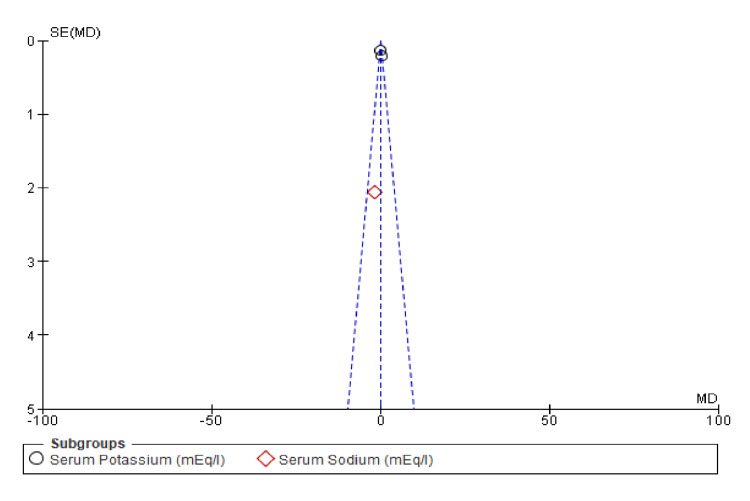
Funnel plots of electrolyte abnormalities showing confidence interval and Mantel–Haenszel ratio for the combined diuretic and placebo groups

The funnel plot illustrated in Figure 12 shows that the studies on serum potassium and serum sodium are symmetrically distributed around the mean difference. This symmetry suggests that there is no significant publication bias or small-study effects in the included studies regarding electrolyte abnormalities in the comparison of the combined diuretic group with the placebo group.

**Figure 13:**
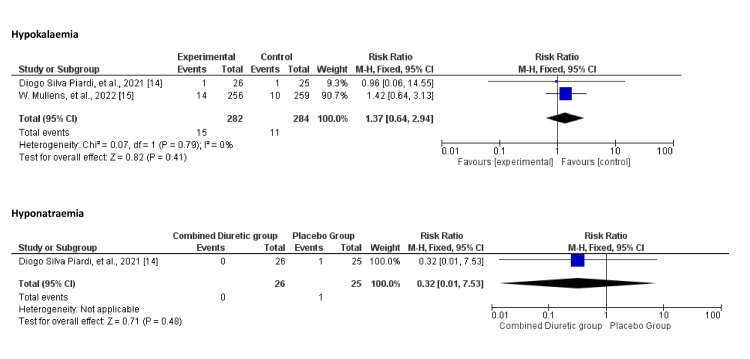
Forest plot for hypokalaemia and hypernatremia showing confidence interval and Mantel–Haenszel ratio for the combined diuretic and placebo groups

The analysis illustrated in Figure 13 did not show a statistically significant difference in the risk of hypokalaemia or hypernatraemia between the combined diuretic and placebo groups.

**Figure 14:**
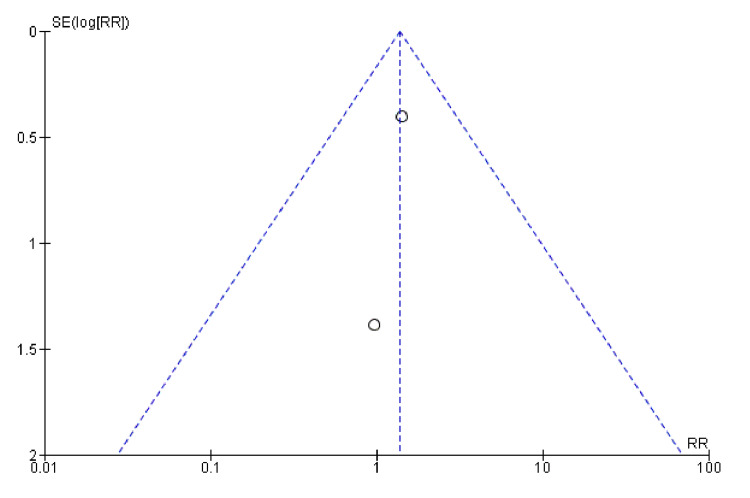
Funnel plot for hypokalaemia showing confidence interval and Mantel–Haenszel ratio for the combined diuretic and placebo groups

**Figure 15:**
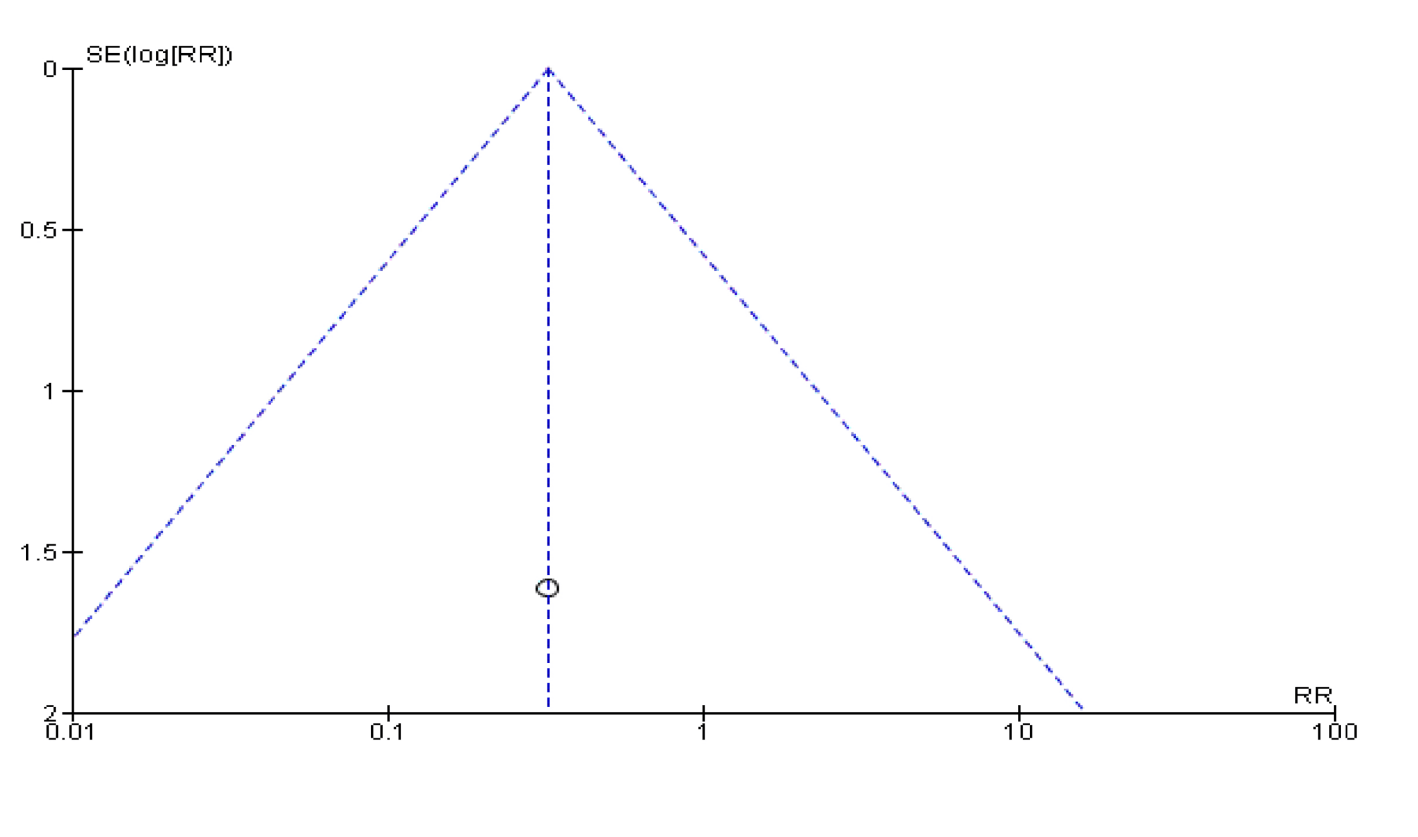
Funnel plot for hyponatraemia showing hypernatremia showing confidence interval and Mantel–Haenszel ratio for the combined diuretic and placebo groups

In Figure 14, the open circles (studies) are spread around the combined-effect estimate line for hypokalaemia, showing some symmetry. However, there are fewer red diamonds (studies) for hypernatraemia, and they appear more dispersed, which might suggest variability or potential bias (Figure 15).

Renal Function

**Figure 16:**
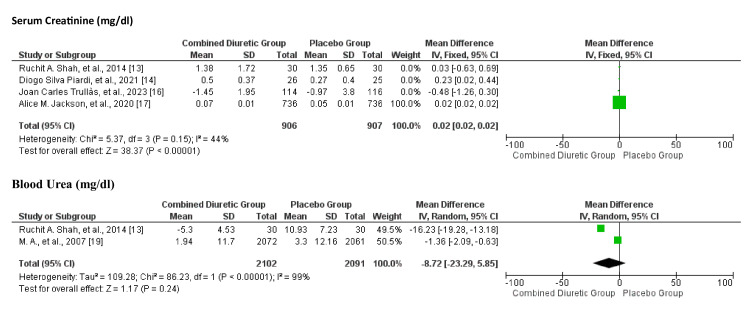
Forest plot for renal function showing confidence interval and Mantel–Haenszel ratio for the combined diuretic and placebo groups

**Figure 17:**
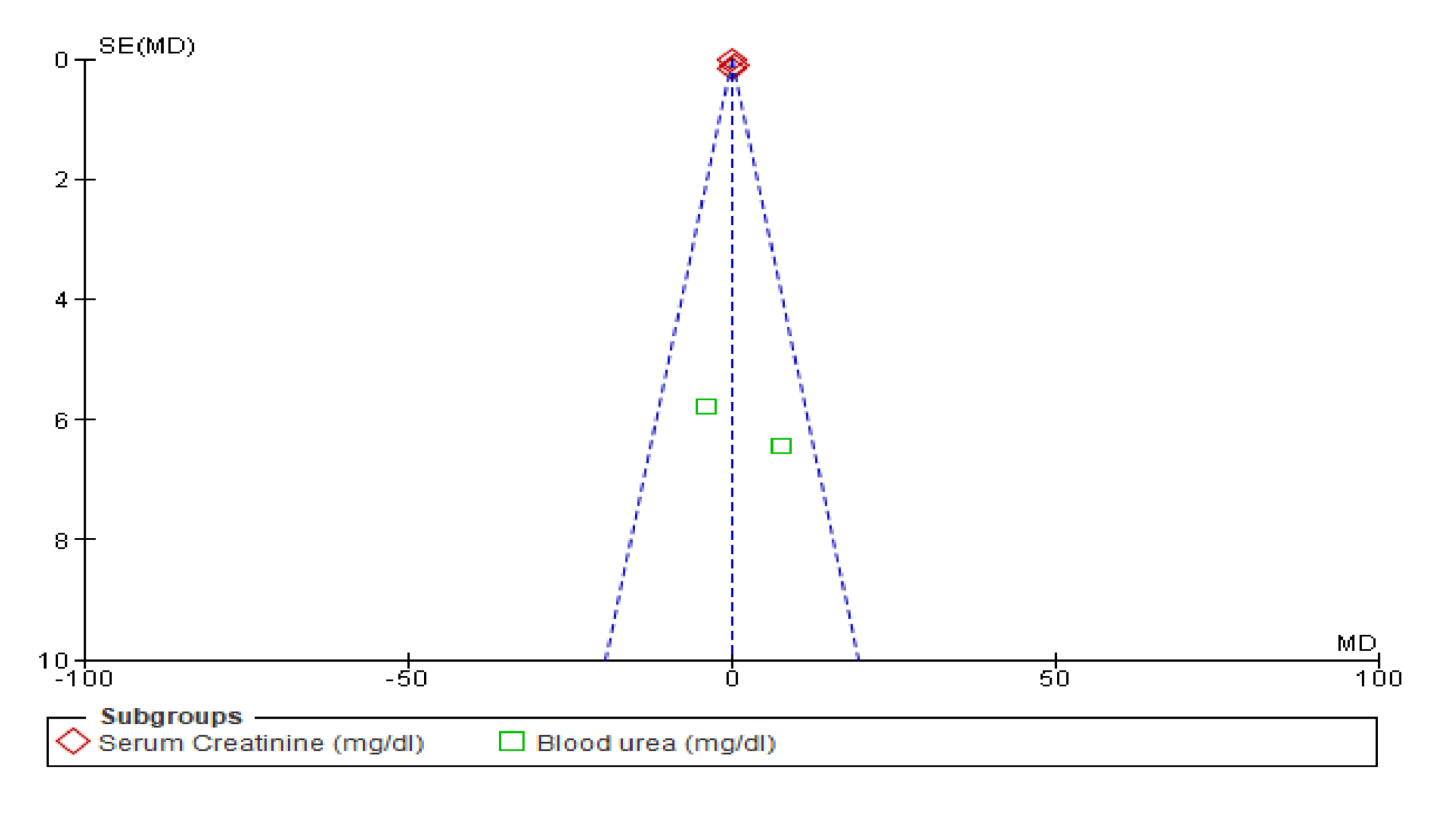
Funnel plot for renal function showing confidence interval and Mantel–Haenszel ratio for the combined diuretic and placebo groups

Figure 16 suggests that the use of combined diuretics does not significantly impact serum creatinine or blood urea levels compared to placebo. Heterogeneity was moderate for both outcomes, suggesting some variability among the included studies. However, higher rates of worsening renal function were observed with HCTZ and high-dose FSM than with placebo. Dapagliflozin showed a slight increase in creatinine levels compared with placebo. Figure 17 provides a visual assessment indicating that the studies on renal function (serum creatinine and blood urea) do not exhibit significant publication bias.

Adverse and Serious Adverse Events

**Figure 18:**
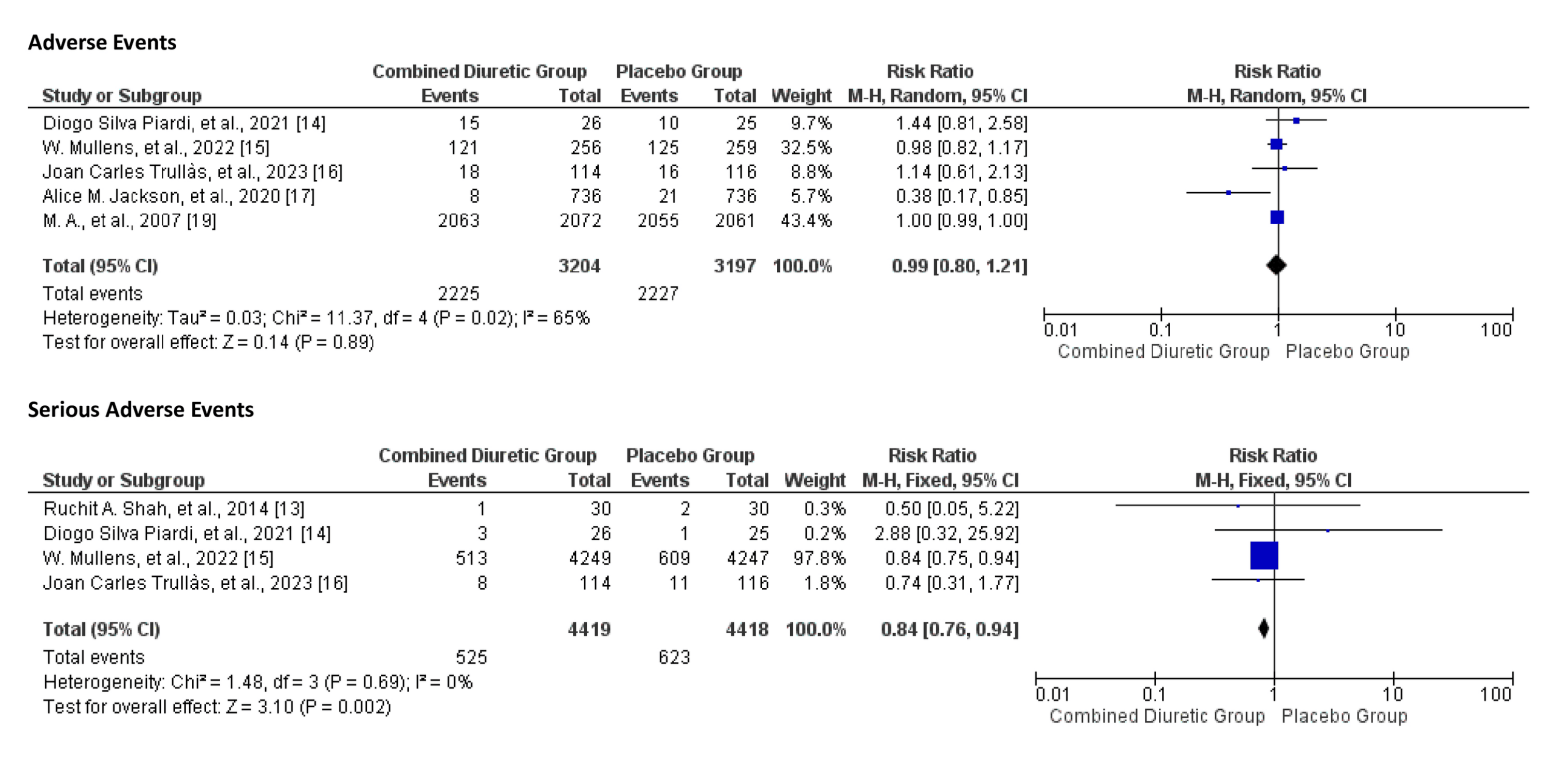
Forest plot for any adverse events and any serious adverse events for the combined diuretic and placebo groups

Adverse events varied among the studies. Some studies reported higher incidences of specific events such as ventricular arrhythmias, haemodialysis, and cardiovascular adverse events. Figure 18 illustrates the risk of adverse and serious events between the combined diuretic and placebo groups. The pooled data indicates that combined diuretic therapy does not significantly affect the risk of adverse or serious adverse events compared to placebo. The overall effect estimate is close to 1.00, with no statistically significant differences observed. The I^2^ value for the groups was 65%, and the chi-square value was 11.37.

**Figure 19:**
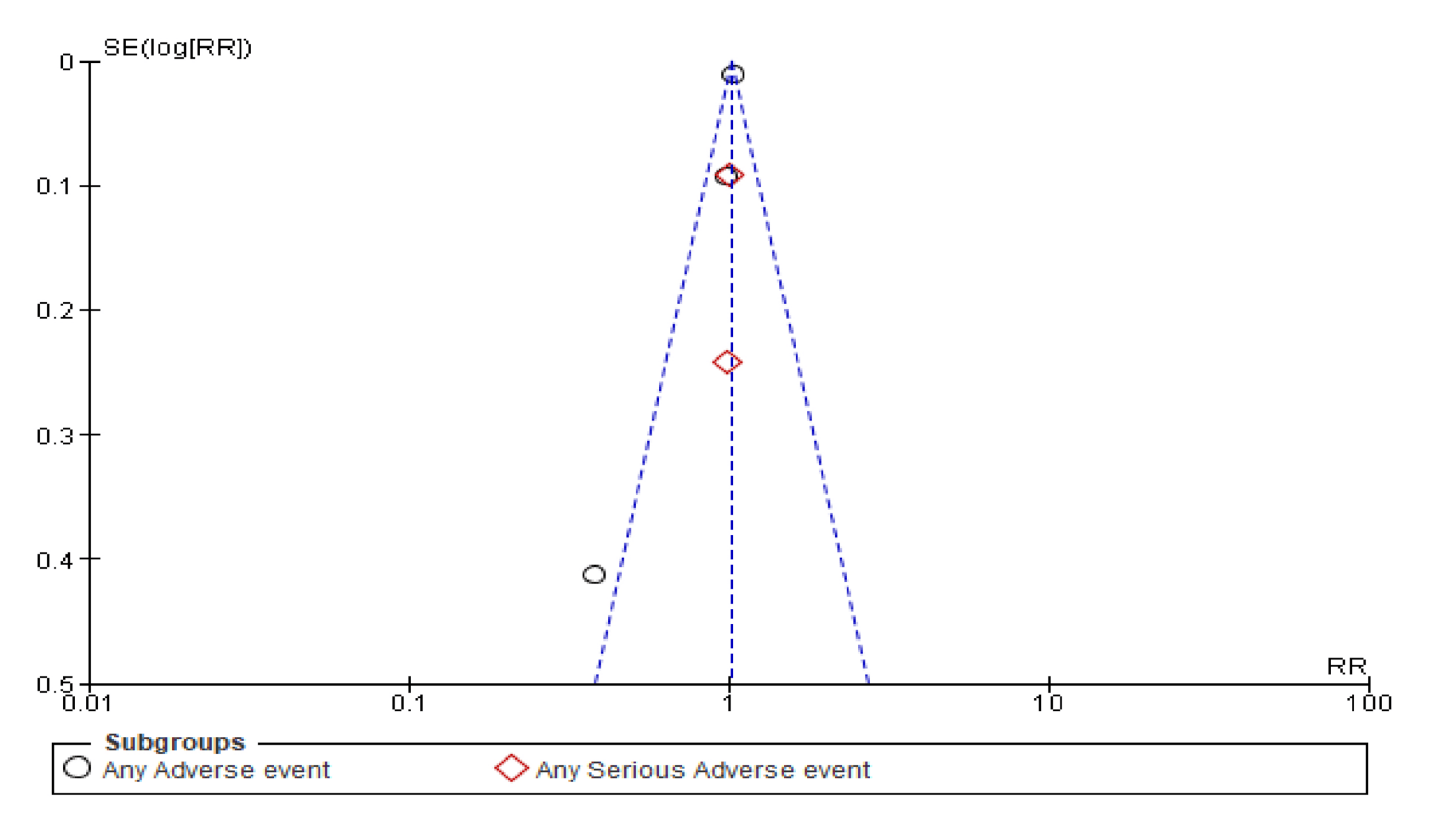
Funnel plot showing results for adverse events and serious adverse events for the combined diuretic and placebo groups

Figure 19 shows that the studies on "any adverse event" and "any serious adverse event" are symmetrically distributed around the mean difference. The presence of one outlier for "any adverse event" could suggest some publication bias or variability in study results, but the general symmetry suggests that significant bias is unlikely.

Subgroup analysis or senstivity analysis of the studies

Subgroup analyses were inadequate in the reviewed studies, but few reports provided insights. Shah et al. assessed serum sodium, potassium, and creatinine blood urea levels across different infusion methods (infusion + dopamine, bolus, infusion alone) [13]. There were no statistically significant differences between the treatment groups. Jackson et al. performed subgroup analyses of patients taking different dosages of loop diuretics and found that dapagliflozin improved symptoms across all dosage groups [17]. Additionally, Piardi et al. found no significant differences in the HCTZ and placebo groups while comparing creatinine levels, dyspnoea scale, congestion score, hypernatraemia, and hypokalaemia. Recently, Mullens et al. compared renal safety parameters between acetazolamide and placebo groups. The results revealed a slightly adverse renal outcome in the former group. These findings highlight the need for customised treatments based on individual patient characteristics and responses.

This systematic review highlights the effectiveness of combination diuretic strategies in managing fluid overload in patients with HF. Studies have consistently demonstrated that various combinations, such as metolazone (MTZ) with FSM, FSM with quinethazone, and bendroflumethiazidewith bumetanide, significantly improved diuresis and weight loss. Refractory fluid overload in HF, characterized by decreased diuretic responsiveness, inadequate sodium and water excretion, and fluid accumulation in the body, remains a significant challenge in managing HF, despite considerable advancements in therapeutics. The combination of MTZ with FSM or empagliflozin showed promising potential for improving symptom management.

Discussion

In this systematic review and meta-analysis, numerous studies explored the effectiveness of combining diuretics to alleviate HF symptoms. The findings from Konstam et al. on tolvaptan and recent trials by Biegus et al. on empagliflozin highlight the rapid advancement of combination therapies [18-19]. SGLT2 inhibitors are increasingly used in HF and have been shown to have diuretic effects. Biegus et al. found that the use of empagliflozin in HF patients resulted in more decongestion, in addition to a reduction in HF hospitalisations and mortality [18]. This aligns with the findings of the DAPA-HF trial, which also demonstrated significant reductions in cardiovascular deaths and worsening HF events with the use of dapagliflozin, another SGLT2 inhibitor [17]. The consistent efficacy of SGLT2 inhibitors across different studies underscores their potential as standard components of combination diuretic therapy for patients with HF.

Similarly, Felker et al. indicated that tolvaptan can be effective for decongestion and alleviation of symptoms in patients with AHF [20]. Tolvaptan is the only clinically approved medication for the treatment of dilutional hyponatraemia and has been shown to improve dyspnoea and oedema while mitigating adverse effects. Tolvaptan can be useful as a diuretic in decongestion, although it may cause side effects, such as activation of the renin-angiotensin-aldosterone system, electrolyte disturbances, and worsening renal function. This study is comparable to one of the RCTs included in this review, the EVEREST trial, which showed that tolvaptan improved congestion symptoms without significantly affecting long-term mortality and morbidity [19].

Comparisons between different diuretic combinations revealed varying levels of effectiveness. For example, the combination of FSM with MTZ was shown to be highly effective in resolving oedema [16,21], whereas agents such as empagliflozin demonstrated superior weight loss and congestion relief compared to placebo [18,22-24]. These findings are similar to those of earlier studies that demonstrated more effective diuresis with combination regimens [21-22,25].

Rasoul et al. performed a meta-analysis on several RCTs involving patients with acute decompensated HF treated with intravenous loop diuretic infusion and boluses. This study demonstrated that there was little to no difference in mortality between the two groups and that the net weight loss was slightly higher in the continuous intravenous infusion group [26]. Additionally, this also study also suggested that there was not much difference for the hospital stay duration and hospital readmission rate between the two groups. Similarly, there was no statistically significant difference between the groups regarding electrolyte imbalance and complications such as ototoxicity, hypotension, and acute renal impairment. Another study by Karedath et al. showed that all-cause mortality was higher in patients group receiving continuous infusion of diuretics compared to bolus injection group; however, this difference was statistically not significant [27]. There was no significant between the two groups regarding patients’ length of hospital stay. The incidence of hypokalaemia was lower in the bolus injection group than the infusion group.

Safety concerns in combination with diuretic therapy remain a significant issue. The analysis of adverse events in this study indicated that while combination diuretic therapy did not significantly increase the overall risk of adverse events compared with placebo, specific adverse events were noted. The risks of hyponatraemia and hypokalaemia have been evident in several studies. However, individual studies have shown variability in outcomes. Piardi et al, reported an increased risk of hypokalaemia in patients receiving HCTZ than in those receiving placebo [14]. Similarly, Trullàs et al. found a higher incidence of hypokalaemia in the HCTZ group [16]. Additionally, Mullens et al. reported higher rates of renal impairment in patients treated with acetazolamide compared to placebo [15]. These findings are critical, as they underscore the need for careful monitoring of electrolytes and renal function during combination diuretic therapy. Therefore, while combination diuretic therapies offer significant benefits in managing fluid overload in patients with HF, their use must be carefully balanced with potential risks.

Tailored protocols considering patient-specific factors such as baseline renal function, comorbidities, and previous diuretic response are therefore key to optimizing therapy and reducing adverse outcomes. Mullens et al. highlighted the need for careful monitoring of renal function and electrolytes when using high-dose diuretics [8]. Additionally, since a significant proportion of hospitalized patients with HF are discharged with residual congestion, there is still a need for future research to explore optimal diuretic combinations and dosing strategies to enhance the efficacy and safety of HF management. By integrating combination diuretics into a comprehensive care plan and addressing the individual needs of patients, clinicians can improve outcomes and enhance the quality of life of those living with HF.

Clinical implications

The findings of this systematic review and meta-analysis suggest that combination diuretics have important clinical implications. Healthcare providers managing patients with HF should weigh the risks and benefits of combination diuretic therapies. These findings also highlight the need for individualized treatment approaches based on patient characteristics and responses. This aligns with the recommendations of the American Heart Association and the European Society of Cardiology, which emphasise personalized treatment plans in HF management [4]. By considering individual patient factors such as age, comorbidities, and baseline renal function, clinicians can optimize therapeutic outcomes and minimise adverse effects.

Study strengths and limitations

The strengths of this systematic review include its comprehensive search strategy, inclusion of RCTs, and rigorous quality assessment of the studies. Methodological rigor ensures that the findings are robust and reliable. Additionally, by reviewing the years of research, this systematic review not only offers valuable insights into effective treatment strategies but also indicates the need for tailored methods in clinical practice. Nevertheless, there were limitations, such as the heterogeneity of the included studies, variations in diuretic combinations and dosages, and potential biases in some studies. In addition, the lack of long-term outcome data in some studies limits the ability to draw definitive conclusions regarding the sustained efficacy and safety of combination diuretic therapy. Future research should address these gaps by conducting large-scale long-term studies to better understand the chronic effects of these therapies.

## Conclusions

While combination diuretic therapies offer significant benefits in managing fluid overload in patients with HF, their use must be carefully balanced with potential risks. This systematic review underscores the importance of personalized medicine, rigorous monitoring, and ongoing research to optimize HF treatment. By integrating combination diuretics into a comprehensive care plan and addressing the individual needs of patients, clinicians can improve outcomes and enhance the quality of life for those living with HF.
